# On the Pathogenesis and Treatment of Sterility in the Human Female

**Published:** 1869-11

**Authors:** Wm. C. Roberts

**Affiliations:** New York


### ON THE PATHOGENESIS AND TREATMENT OF STERILITY IN THE HUMAN FEMALE.

By WM. C. ROBERTS, M.D., of New York.

In May last, I read before the N. Y. Academy of Medicine, a paper on the causes of sterility in either sex, based mainly upon physiological relations.

Subsequently, Dr. Kammerer read a paper on the pathological conditions causing sterility, based upon a view of 408 cases, 201 of which had occurred in his own, and 207 in clinical practice (*Trans. N. Y. Ac. of Med.*, vol. 3, p. 7). These cases, which embraced the numerous descents, deviations, and diseases to which the uterus and its appendages are liable, were treated with more or less success, according to the usual methods; the proportion of success, so far as the removal of the sterility was concerned, being about one-third of the cases treated. One case in particular, however, deserves to be mentioned (No. 12); that of a woman whose inner uterine orifice was dilated at one session just after menstruation, and who conceived, after a barrenness of four years, immediately after; whether *propter hoc*, cannot, perhaps, be exactly determined. The physiological causes of sterility are not alluded to in Dr. K.'s paper, unless, indeed, the dilatations of the uterine orifices were intended to allow of an easier admission of the spermatozoa to the cavity of the uterus; and the intra-uterine injections employed to remove the noxiousness of the secretions.

In the paper which I read before the Academy, I showed:—

1. That the generative apparatus of both sexes must be in a healthy condition. In the male, the penis must be capable of erection and ejaculation, and of emitting healthy semen.

In the female, the uterus and its appendages must exist, and be perfect; the ovaries contain fecundible ova; the tubes be pervious; the lining membrane of the cavity and neck healthy; and the os uteri, externum and internum, hymen, and vagina, pervious.

2. That it was by no means necessary that there should exist, at the moment of coitus, any orgasm on the part of the female, or a complete introition of the male organ intra vaginam; a very slight peri-vulvular congressus depositing the semen upon the vulva, sufficing for impregnation.

3. The ripe graafian vesicle, secreted either just before, during, or after menstruation, and even, though not often, during the intermenstrual period, must, in some part of its course into the uterus, come into direct and immediate contact with one or more living spermatoza, in order to be fecundated.

4. Semen contains, as its most important constituent, animalcules, spermatic cells, zoospermes, spermatozoa, or zoids, as they are variously called according to the idea which is formed of their nature. In the field of the microscope, they are seen to move about with varying activity, and whether or not they be endowed with true vitality, life, or be or be not organized animals, which last seems generally now to be believed, their volition is seemingly directed by instinct, towards, and in spite of all obstacles, the ovum which they are to impregnate. In a natural temperature, they live for 48 or 72 hours; are found living, even in the cadaver, after 24 hours, and in bitches have been seen to move seven or eight days after copulation. Acids, urine, electricity, strychnia, narcotics, and certain vagino-uterine secretions destroy them; but of this last hereafter. Probably they are reproduced; they are certainly nourished: strange creatures, which, by union with an ovule, are capable of communicating to it, not only the physical resemblance, but the temperament and constitution of the parent. They appear in the semen at puberty; are found afterward at all periods of life, and in men of advanced age (82) have been found as numerous as in the adult.

5. The material contact of the semen and the ovule, both animated by their vitality, and perfect in themselves, is the essential condition of fecundation, and the intimate fusion of these two elements is alone capable of giving birth to the new being. *If any obstacle* impedes the immediate contact of the two germs, conception on the part of the female is impossible. Upon an accurate knowledge in regard to these causes depends the successful treatment or cure of sterility.

6. The *aura seminalis* alone is insufficient. Filtered semen is equally so. No part of the semen but the animalcules suffices.

7. The fecundating power of the animalcules seems connected with their vitality, for it diminishes, and is completely extinguished with their movements. Semen is infecundible without living spermatozoa; and it is certain, however they enter it, that they get within the vitelline membrane of the ovule, and have been seen in immediate contact with the yolk, when they part company and disappear by liquefaction.

8. The merest drop of a high dilution of the semen of a frog, directly applied to the egg of the female, suffices to fecundate; but more than one spermatozoid is required. Two hundred and fifty-five, in the experiments of Prevost and Dumas, impregnated sixty eggs out of three hundred.

9. Neither the movement of the vibratory cilia, nor an aspiratory spasm, nor capillarity, can account for the progression of the spermatoza. It is to their own motility, and to their power of overcoming obstacles, that this is wholly due. The passage of the spermatoza from the uterus into the tubes occupies 8 to 10 hours; arrived at the free extremity of the tube, they reach to, or upon the ovary, by means of the fimbriæ which unite the *pavillon* to that organ. If there they meet with a mature ovule, fecundation may result. Twenty or thirty minutes are required to enable them to enter the uterus. The tubes take from two to six days to transmit the detached ovule to the uterus, where, if previously fecundated, or when fecundated it stops and is developed, imbedded in decidua. If not, it escapes with the decidua in 10 or 12 days, or at the end of menstruation. The period most favorable for impregnation, then, is immediately before, or during, or soon after menstruation ceases. The flow of menstrual blood does not impede, but rather accelerates the progress of the spermatozoa. But how are we to account for fecundation during inter-menstrual periods, unless we suppose that coition hastens the development and detachment of a mature ovum? Fecundation and coition are separated for at least the time which is required for the spermatozoa to pass through the uterus and tubes, and reveals itself by no special signs. A single act of coitus may suffice for impregnation, of which many instances are known. If, now, we attempt to assign the causes of infecundity from a physiological point of view, we shall find that men are infecunds because they are impotent or aspermatic, *i. e.*, incapable of erection or ejaculation: and even when capable of emission, are as aspermatozoic, that is, secrete a semen or fluid which contains either no or no living spermatozoa. Eunchs possess for a while an incomplete power of erection and an ejaculation which must be aspermatozoic, which, for the lack of the stimulus of venereal appetite, they gradually lose. Impotence is not necessarily associated with either aspermatism or aspermatozoa. It is sometimes purely nervous, and when cured, the power of fecundation may exist or return. It occurs among the newly married and in the old. But very old men are not necessarily infecund, as we have seen, and the case of old Parr is in point. The only way in which aspermatozoa can be positively ascertained, is by submitting the semen to the microscope soon after its emission.

But it is chiefly with infecundity in the female that we are concerned, and allowing that there is no fault in the generative faculty of the male, it behooves us to enquire into its causes. Admitting that no physical defect of organism occur in her, and that, as we so often see, she is robust, healthy, menstruates more or less perfectly, and is free from organic uterine malformation or disease, why is it that the spermatozoa do not reach and fecundate her ova? Ill-health may, I think, possibly prevent this from occurring, by impairing the fecundity of the ova, or faults in the ovary or ova may have a similar effect. Dysmenorrhœa, though often associated with infecundity, does not necessarily cause it, and the reason of the association is probably purely a mechanical one. The cause which prevents the easy escape of the menses, and renders it painful, may equally prevent the access of spermatozoa to the uterus, tubes, and ovaria. To these we shall presently advert.

But these are not the only causes for infecundity on the part of the woman. There is another and a principal one, to which passing allusion may be found in authors, but it has by no one been so markedly assigned and scientifically considered as by Donné, and our countryman Dr. Sims, to whom Surgery and Science are alike both deeply indebted.

I allude to the *destruction of the spermatozoa*, by the vitiation or peculiar constitution of the vagino-uterine secretions, by which fecundation is rendered impossible.

In Donné's "Cours de Microscope," etc., the work of a zealous, cautious, and candid observer, we find much that is interesting and important on this subject. Acetic acid instantly kills the spermatozoa, but leaves them perfectly intact for years. Blood and milk exert upon them no deleterious influence, saliva kills them rapidly, urine instantly. Pus and the *muco-purulent matter of uterine leucorrhœa* does *not* affect them by its contact. They live perfectly well in the mucus secreted by the vagina in a normal state, which is slightly acid; but, and this observation is most important, the ascidity of the mucus secreted by the vagina becomes such, in some circumstances, as when there is congestion, acute irritation, or inflammation of this organ, that the zoospermes *seem unable* to live in it more than a few moments. He has even seen them, particularly, give no sign of life, in less than two minutes, in the vaginal mucus of a woman of 22, affected with an extremely acid discharge. "Can this, then," Donné says, "be considered as the cause of sterility in some women?"

*Vaginal* mucus is white, opaque, creamy, not viscid, and always *acid.* *Uterine* mucus, on the contrary, is transparent, stringy, tenacious, like albumen, sometimes clouded with purulent matter, but always *alkaline*, turning litmus paper blue, whilst the mucus of the vagina always reddens it.

The action of this (uterine) mucus on the animalcules varies according to circumstances. Generally the spermatozoa brought into contact with uterine mucus do not suffer. But *certain kinds of uterine mucus* kill the animalcules with the greatest rapidity. Nor is this mucus distinguished from others by any appreciable characteristic, microscopic or otherwise, being either pure and transparent, or opaque. An excess of alkali seems to be the only probable cause of its deleteriousness, litmus paper becoming instantaneously intensly blue.

No possible means of ascertaining the fact seems to exist, except that of submitting the spermatozoa to the action of uterine mucus of various kinds or qualities. "Do not," says Donné, "the facts related lead to the belief that alterations of the vaginal and uterine secretions play an important part in causing sterility, by killing the fecundating liquid; and is not some light thrown on its hitherto obscure causes, and a suggestion made of a rational and efficacious remedy?"

It is but doing simple justice to our countryman, Dr. J. M. Sims, to say, that he is the first among us to revive those ideas, and give to them a practical application (On Mic. in Diag. and Treat. of Sterility, *N. Y. Med. Jour.*, Jan., 1869). In this paper he lays it down: 1st, we must have spermatozoa in the semen; 2d, they must enter the utero-cervical canal; 3d, the state of the secretions must be favorable to their vitality. In the absence of the second of these conditions only is any operation to be thought of. How are these facts to be ascertained? By examining the condition of the vaginal and uterine secretions after coition. A little of each is to be withdrawn with a syringe, and placed under the microscope; and to do this accurately, the fluid must be retained for some time after in the vagina. He thinks the best period for making the investigation is the fifth or sixth day after the menstrual flow. Dr. Sims, in one respect, differs from Donné. He says, "the vaginal (normal) mucus, by its natural acidity, kills very quickly *every* spermatozoon, and seems to be a perfect poison for the superabundant ones." If this were true, fecundation would very seldom, almost never, occur. Donné, more correctly, I think, says, its slight natural acidity is not noxious to the spermatic animalcules, *but is only so when excessive.* The cervical mucus is to be carefully separated and distinguished from the vaginal, and withdrawn with a syringe for examination. Dr. S. thinks it possible to obtain a second specimen from higher up the canal, or even from within the os internum, which I should think would be difficult, and finds in the one sometimes living, in the other dead spermatozoa. Donné does not carry his researches so far. He is content to take the mucus which hangs out of the os externum, or can be withdrawn from within the neck. Dr. Sims thinks that if the cervical secretion contains little opaque spots of milky whiteness, and when it is very thick and albumino-purulent (as also when perfectly clear), it is poisonous. Donné's observations *generally* (p. 293) oppose this assertion:—*A certain quantity of mucus is necessary to produce this effect*, which cannot be told from its natural or microscopic appearances—too *alkaline* (?) if uterine; *too acid* if vaginal. Be all that as it may, there is a peculiar condition of either of these secretions, whatever it be which does kill the spermatozoa, and occasion sterility; and the great point is to remedy it. Dr. Sims justly says, it is not every woman who has dysmenorrhœa (and I add, or leucorrhœa), who is sterile, nor every man who may be vigorous and enjoy good health, who is capable of procreation. He has known half-a-dozen husbands—in one place he says many—whose semen had no spermatozoa. Dr. Sims' paper proves and he frankly acknowledges, that the operation of incising the cervix is seldom necessary or proper, often quite uselessly performed; and he no longer thinks that the most common obstacle to conception is a more or less contracted utero-cervical canal (p. 24, lib. cit.) I quite agree with Dr. S. that the necessary investigations into this interesting and practical subject, by which alone a true and scientific knowledge and basis of action can be obtained, involve neither indecency nor sacrifice of self-respect on the part of either surgeon or patient.

If, then, it should be asked, what necessity then exists for dilating the two orfices and neck of the uterus, we answer, 1st, because of its allowing a more easy escape for the menstrual blood; and 2d, because it allows an easier access to the spermatozoa. Although neither an aggravated dysmenorrhœa, nor a very contracted os uteri, internum or externum, are necessarily fatal to, they are unfavorable to impregnation, and should, as a part of the treatment, be remedied. I cannot, for my own part, imagine that flexions, however great, of a part so short and, so flexible as the neck of the uterus, which are so readily restored by the introduction of a long and suitably-sized speculum, and which, moreover, I so seldom encounter, can offer any serious impediments to fecundation. But the contractions of the orfices are real and unmistakable, often obstinate, and contribute, I doubt not, to this result; but even then very partially, for, as Dr. Sims justly observes, cases are recorded where conception occurred when the os barely admitted a small-sized probe, and that spermatozoa now and then pass along the Fallopian tubes, which ordinarily admit a bristle. It is, then, to the state of the vaginal and uterine secretions, the semen being healthy, that we must look for the great cause of infecundity in the female. The remarks of Joulin on this subject are worthy of repetition:—"The contraction which has its seat at the internal orfice of the uterine neck is one *of the most common causes of infecundity;* and particularly among multiparæ. But women who have borne children sometimes exhibit this disposition, which oftenest coincides with an extreme narrowness of the neck of the womb and imbrication of the folds of the *arbor vitæ.* This cause of sterility which is usually accompanied with the phenomena of dysmenorrhea, was until lately unknown.

"The treatment consists in dilating the constricted region. I prefer a cylinder of prepared sponge, small to begin with. I have obtained 2 successes with this proceeding;" Becquerel 4, and Meisteler 7, out of 9; McIntosh 24 out of 27, 11 of whom bore children; and Corty several followed by conception (410). This certainly shows that, although closure of the uterine orfices does not necessarily prevent, dilatation exerts a considerable influence over the subsequent fecundity of the patient.

"The first sponge," says Joulin, and Corty repeats him, "does not pass through the internal orfice; each one penetrates a little further than the first, and simply dilates the neck; but later, the inner orfice becomes permeable. The sponge applied swells immediately, and the woman can attend to her business without feeling the least inconvenience." This is only partially true. It sometimes produces pain and irritation within 24 hours, and requires removal. I once saw frightful results from the thrusting of a sponge-tent into the uterine cavity. Corty says an interval of 2 or 3 days should elapse between each introduction. Joulin says, if not removed, generally about the 3d day a discharge takes place of a clear, abundant, *fetid* fluid, which disappears on its removal. "I renew" he says, "these applications twice a month—10 days before and after menstruation. When the dilatation is sufficient, I suspend their introduction, and recommence if the stricture tends to reappear, for often the amelioration is only temporary. It supplants the incision, which, unless the sponge-tents are used simultaneously, cicatrizes and contracts, leaving things as they were. It is simpler and less dangerous, and women, at least, if they have not patience to follow out the plan, *are often relieved of their dysmenorrhœa.*" "Uterine deviations," says Joulin, "are not causes of sterility, for the semen need only to be deposited in the vaginal cavity, not projected into the os. Unless, then, when very intense, and the angle of flexure very great, displacements of the uterus do not offer any serious obstacles to conception." Tents of sea-tangle (*Laminaria digitata*) are preferable to prepared sponge, because they can be longer retained without producing irritation and fetid discharge, and do not rot and break; but I do not find them as easy of introduction. Corty repudiates them.

Other means of dilatation are employed. Dr. McIntosh used flexible metallic bougies. Bennet prefers wax or gum-elastic bougies to metallic ones. Simpson uses metallic stems of graduated sizes, left in the uterus for various lengths of time, and sometimes changed daily for days together, or left in permanently. With the gum-elastic French bougie, fine or olive-pointed, varying in diameter above the point (bougie à ventre), all my own successes have been attained. Generally, the point reaches to and strikes firmly against the os internum, and irritates it spasmodically. After a time, if not too large, with a little management, it enters the stricture and passes up to the fundus with little or no pain. The next time, again, perhaps, nothing will pass, and so on, until sufficient dilatation is effected to admit a larger size. Sometimes, on the same day, one bougie, after remaining in a little while, may be withdrawn, and a larger one passed, but not often. Strictures of the os internum uteri, like strictures of the urethra in the male, are irritable and capricious, and must either be coaxed or taken when in good humor. If it is going in, the bougie must pass in quickly, almost precipitately. I think I understood Dr. Kammerer to say that he also made use of steel bougies of graduated sizes. All I have to say about this is, that safe as these may be in skilful hands, and successful—they have not been so in mine—they cause pain, and, I think, excite irritation and spasmodic contraction in the upper os, which makes it difficult to pass them through it; and if pushed in forcibly, might, I should think, do mischief. I can conceive of cases in which it might be necessary to incise the outer os, or to dilate the cavity of the neck with sponge or laminaria, in order to introduce the bougie; but when a hysterotome of any kind, a sponge-tent or slip of laminaria can pass, a slender-pointed, flexible, gum-elastic bougie, I

should think, could be passed also, and with time and patience effect the object. Try to learn the course of the stricture, pass in the bougie in it, firmly and quickly, through the os, *au fond.* If it means to go, it will then, and may be retained for a longer or shorter time. I believe it would be well to leave it in for some hours. If you do not succeed after a short trial, suspend the operation till a later day. If the stricture is, or becomes irritable, the more you try, the less likely you are to succeed. Success is either null or immediate. When complete dilatation has been obtained with a bougie of large size, the passage of it becomes easy, but it must be repeated at intervals; after menstruation; perhaps best in the true inter-menstrual period (10 or 14 days after), for if the woman should happen to have conceived, abortion would almost inevitably follow the introduction of the bougie into the uterine cavity. The bougie should be new, firm, and not too flexible, or it will double up and not pass. Bougies that have been used many times are liable to this objection. If, after withdrawing the bougie, a laminaria tent cou d be introduced, and left *a demeure* some days, it would be well: but it is not easy or always possible, and the bougie alone must be depended upon.

All these operations have for their object the facilitation of the escape of the menses and removal of dysmenorrhœa, and the allowing of an easier and freer entrance to the spermatozoa. But beyond this is the aid afforded to methods intended to effect the change in the uterine secretions necessary to insure the vitality of the spermatozoa. If this cannot be done, our researches and conclusions upon this point in the pathology of sterility will be of no avail. It is to be effected, as far as I know, by cauterization, chiefly with nitrate of silver, introduced in the solid form, or by injection, into the cavity of the neck and uterus; although other substances are employed. Dr. Sims' pamphlet is unfortunately silent on this point. Corty ranks among curable causes of sterility, simple or complicated imperforation, congenital or accidental narrowness of the uterine orifices, the frequent cause of mechanical dysmenorrhœa; and says by dilatation and double incision of the neck, he has obtained unquestionable cures of more than 15 sterile women, whose fecundation followed the treatment in from 3 to 15 months. Flexions, when very decided, are causes of sterility, and incurable when held down by adhesions; and he does not partake of the opinion of M. Joulin, on the small inportance of deviations of the uterus as causes of sterility. He thinks that the glans penis should, at the moment of ejaculation, be opposite to the meatus uterinus; and that even a temporary restoration to the natural position (p. 1008) suffices sometimes for fecundation; and that after this, and after dilatation even, no time should be lost in attempting fecundation, and that certain positions of *the female in coitus* are preferable in some cases to others. Hypertrophy even of one lip, and conicity of the neck, congestion, inflammation, granulations, and fungosities are all obstacles to fecundation. This appears to be also the opinion of Kammerer, and of C. Mayer, of Berlin (1856), whom he cites in his paper.

In Dr. K.'s patients, retroversion (20), anteversion (18), anteflexion, (83), retroflexion (71), hypertrophy (65), small os (24), stricture of internal os (35), were the commonest lesions, and $\frac{7}{8}$ths of the whole number were affected with some form or other of uterine catarrh. If the views which I have herein presented, relative to the occasional and frequent destructiveness of the accompanying secretions to the spermatozoa be correct, some of the sterility in them may be fairly, I think, attributed to it, and some of the successes to the means employed for its removal. Corty thinks that very abundant leucorrhœal secretion may expel the semen from the uterine cavity, when it has once entered, and the very viscid, tenaceous mucus which plugs the os oppose its entrance.* Vaginal, he thinks less injurious than uterine mucus, the latter of which may have a very injurious influence upon the relatively very small quantity of sperm which enters the uterine cavity (1012). He adds:—In a great number of cases the great abundance of the leucorrhœa, and even its purulence, does not prevent fecundation.

Corty differs from Joulin as to the effect of a want of orgasm on the part of the female. He thinks it may occur without being felt or expressed, that it is capable of development and education, and hence fecundation seldom occurs until after some months (12, 18, 24 and 36) of married life; again, in some cases of sterility, coinciding with perfect general health of the genitals, and only attributable to absolute defect of orgasm, even in women very desirous of becoming mothers; or awaking, together with fecundity, after a lapse of years, and thence pursuing the natural course of its evolution; a new lover, or husband perhaps, determining the result. But it is equally certain that many entirely cold and passive women are extremely fecund. Sterility, on the other hand, may depend upon a totally opposite condition. Menorrhagia (a precocious abortus),

* Sometimes the semen is all instantly thrown off by the vagina, by too soon rising after coitus (Sims 12).

or inter-menstrual menstruation, by its tendency to reproduce itself when the ovum is recently fecundated, becomes an obstacle to gestation. The number of causes, single or allied, upon which sterility may depend, should certainly render us sober of promise to women in whom it is only relative, and affords hope of recovery; while the very frequent realization of that hope, under well directəd treatment, should warrant, I think, a patient and continued effort on the part of both patient and physician.

Among the chief means of correcting the morbid conditions of the uterine mucous membrane upon which noxious secretions may depend, may be said to be cauterizations of the mucous membrane of the neck and cavity, and certain intra-uterine injections. The caustic most commonly in use is nitrate of silver, used externally to ulcerations and abrasions of the neck, and internally applied to the mucous membrane of the neck and cavity, to repress granulations and fungosities, to diminish hypersecretion, and by modifying the morbid condition of the glands which produce leucorrhœa, change the character of the secretion itself into one less injurious to the vitality of the spermatozoa. Upon this subject M. Corty says:—"The cauterization may be performed, after dilatation, with a camel's hair brush, but as this is an unenergetic method, the idea has arisen to introduce the solid nitrate and apply it directly over the surface."

Various porte-caustics have been devised for this purpose, intended to secure the caustic from being broken off within the cavity. "But" says M. C., "having myself met with the accident and satisfied myself not only of its harmlessness, but of its happy results, I fix a piece of solid nitr. of silver, of suitable length, in an ordinary platinum porte-nitrate with a long handle; place the patient in supination; introduce a wooden speculum; examine very gently with a sound the direction of the utero-cervical canal, and immediately after carry the crayon of nitr. silver even into the uterine cavity." This is easily said, but is it quite as easy to do? M. C. continues: "Instead of endeavoring to withdraw it intact, I use, on the contrary, all my endeavors to precipitate it, to break it off, which is not very easy to do, and I abandon it in this cavity (p. 265). The vagina is then tamponed with a cloth, wetted in a solution of salt in water, sustained by a second one, and the speculum withdrawn." "I can say," he adds, "that I do not know any more heroic means that this *little* operation, which it is not often necessary to repeat, in the treatment of obstinate leucorrhœa. I have not observed serious accidents to follow it. Once only I saw atrocious pains, not yielding to baths, antispasmodics, nor narcotics." The neck having swelled, and the os being occluded, he incised it some hours afterwards, to facilitate the expulsion of the mucus and caustic itself. In all the other cases, the pains, "even very strong in a small number of women," have always yielded, however violent, to general and local antispasmodics, and to cool hip baths, with continuous vaginal irrigations, prolonged if necessary for several hours.

Those of my readers who may feel disposed to adopt this heroic and comparatively innocuous and "incomparable" little remedy, 619, will do well to consult M. Corty's Traité Prat., &c., Paris, 1866, p. 264 & 7, 618. It is to be employed only, be it remembered, when the passages are freely pervious, and there exists neither metritis, perimetritis, ovaritis, flexion, deviation, stricture, nor constriction of orfices.

M. Corty is not in favor of intra-uterine injections. He says that they are the most dangerous that can be employed, and has seen them instantly followed by very formidable accidents, to say nothing of the possibility of their passing through the tubes into the peritoneal cavity and giving rise to fatal peritonitis. M. C. uses no other fluïd than pure water, and even then only where there exists a perfect facility for the escape of the injected fluids.

Dr. Jas. Kammerer, of this city, whose paper on Uterine Catarrh, reprinted from the *Am. Journ. of Obs.*, vol. ii., No. 2, August, 1869, I have just been favored with, is a warm advocate, on the other hand, in their behalf, and uses them constantly with safety and success. When catarrh is accompanied with true angular anteflexion, the posterior lip of the uterus must be incised by Emmet's operation, to straighten the canal and provide for a free exit for the catarrhal secretion; and extreme smallness of the external os requires a bilateral incision. The patient being placed in a recumbent position, a sound is introduced into the uterus, and the depth, capacity of the cavity, and the mobility of the organ accurately measured and ascertained. A cylindrical speculum is then introduced into the vagina. In order that an entire permeability of the cervico-uterine canal shall exist before the injection is attempted, Dr. K. is careful to dilate it by means of a series of sounds, four in number, of varying sizes, made of copper or German silver, of which exact figures are given on pp. 14 and 15 of the pamphlet alluded to. These are successively introduced through the speculum, until full dilatation of the canal is obtained; after which injection and escape of the injected liquids are easy and safe. After washing out the cavity of the uterus with a long-nozzled India-rubber bag syringe, the appropriate remedy, in liquid form, is injected. These are chromic acid, Lugol's sol. of iodine, dilute or concentrated, and carbolic acid diluted, and also sulph. zinci, 10 grs. to the ounce. Of carbolic acid as an intra-uterine injection, I find no notice in the three French works I have consulted, Joulin, Corty, and Becquerel, and do not know with whom it originated.

Dr. Squibb, in a recent pamphlet, speaks of it as a mild local anæsthetic, useful in cystitis and leucorrhœa, and says that its use has often been favorably noticed in the foreign journals. Weak solutions of these dilute substances may be freely injected, raised to a certain temperature, not cold; but of the concentrated, 10 to 20 drops is the most that can be safely injected, the dilute being in ordinary cases the most prudent. Caution and skill are necessary; for Dr. K. himself allows (p. 23) that accidents may occur occasionally, notwithstanding the most careful observance of the rules he has laid down (p. 12). Dr. K. observes that he has often cured patients of their catarrh, as well as their sterile condition; but the peculiar morbid action of the secretions upon the spermatozoa, to which, in this paper, I have endeavored to direct attention, is not, so far as I have observed, alluded to. Dr. K. remarks that a new cause of sterility, hitherto little attended to, may be a destruction of ciliary uterine epithelium from the too powerful application of escharotics. The direction of the cilia, however, is from, and not toward the uterine cavity. There can be no doubt of the safety and efficacy of intra-uterine injections in Dr. K.'s experienced hands, and many cases are cured by it no doubt, which would resist any other mode of treatment. Dr. K. looks upon uterine catarrh as the most frequent cause of sterility in the female, having met with it in 342 cases out of 408, but without assigning any reason.

The womb in the present state of uterine therapeutics, must be looked upon as a meek and long-suffering organ, which endures with wonderful patience the rough handling, the burnings, slashings, pokings, and scrapings it receives at the hands of the gynæ-atrics. Still gentleness and caution are necessary; for it does not *always* submit to them without resistance, remonstrance, or recalcitration.

If these remarks shall conduce to a better study and understanding of the causes of sterility, and a more successful treatment for its removal, my object in writing them will be happily attained. The action of the vagino-uterine secretions upon the spermatozoa, will soon, I have no doubt, become an interesting and important specialty, and, I trust, will receive speedy and due attention.—*N. Y. Med. Record.*

